# Delineating Repetitive Behavior Profiles across the Lifespan in Fragile X Syndrome

**DOI:** 10.3390/brainsci10040239

**Published:** 2020-04-17

**Authors:** Debra L. Reisinger, Rebecca C. Shaffer, Nicole Tartaglia, Elizabeth Berry-Kravis, Craig A. Erickson

**Affiliations:** 1 Division of Developmental and Behavioral Pediatrics, Cincinnati Children’s Hospital Medical Center, Cincinnati, OH 45229, USA; debra.reisinger@cchmc.org (D.L.R.); rebecca.shaffer@cchmc.org (R.C.S.); 2 Department of Pediatrics, University of Cincinnati College of Medicine, Cincinnati, OH 45267, USA; 3 Developmental Pediatrics, Children’s Hospital Colorado, Aurora, CO 80045, USA; nicole.tartaglia@childrenscolorado.org; 4 Division of Developmental Pediatrics, Department of Pediatrics, University of Colorado School of Medicine, Aurora, CO 80045, USA; 5 Departments of Pediatrics, Neurological Sciences, Biochemistry, Rush University Medical Center, Chicago, IL 60612, USA; Elizabeth_Berry-Kravis@rush.edu; 6 Division of Child and Adolescent Psychiatry, Cincinnati Children’s Hospital Medical Center, Cincinnati, OH 45229, USA; 7 Department of Psychiatry and Behavioral Neuroscience, University of Cincinnati College of Medicine, Cincinnati, OH 45267, USA

**Keywords:** fragile X syndrome, restricted repetitive behaviors, gender, autism spectrum disorder

## Abstract

Restricted repetitive behaviors (RRBs) are a core area of impairment in autism spectrum disorder (ASD), but also affect several other neurodevelopmental disorders including fragile X syndrome (FXS). Current literature has begun to describe the RRB profile in FXS up through adolescence; however, little is known about the subtypes of RRBs in adolescents and adults. Further, literature on the RRB profile of females with FXS is limited. The present study examines the RRB profile across subtypes and specific items in both males and females with FXS while assessing for differences based on age, ASD diagnosis and the impact of IQ. Participants included 154 individuals with FXS (ages 2 to 50 years old). Results revealed a peak in RRB severity in FXS between 7–12 years for the majority of RRB subscales with the exception of Sensory-Motor behaviors peaking between 2 and 12 years before declining. Distinct RRB profiles in males and females with FXS emerged in addition to significant overlap among the item and subscale levels of RRBs across gender. Further, an added diagnosis of ASD significantly increased rates of RRBs across all subscale levels, but not necessarily across all items. Lastly, IQ did not solely account for the presence of RRBs in FXS, with Sensory-Motor behaviors being driven by comorbid ASD in males with FXS, and Restricted Interest behaviors being driven by comorbid ASD regardless of gender. These findings build on the current understanding of RRBs in FXS based on gender and comorbid ASD and lay important groundwork for the development of targeted behavioral and pharmacological treatments.

## 1. Introduction

Restricted and repetitive behaviors (RRBs) form a class of behaviors characterized by stereotyped or repetitive movements, inflexible adherence to routines or ritualized patterns of behavior, restricted interests, and unusual sensory interests [1]. RRBs are a core area of impairment in autism spectrum disorder (ASD), but have also been found in other neurodevelopmental disorders, including fragile X syndrome (FXS) [2]. To date, there has been literature suggesting individuals with FXS at the global level exhibit RRBs, with a few studies examining the behavioral phenotype of RRBs in FXS up through adolescence [2,3,4]. Unfortunately, little is known about the progression of RRBs in FXS throughout the lifespan, including limited information on the differences between males and females and the impact of comorbid ASD. The present study is the first to examine the RRB profile in males and females with FXS from early childhood across late adulthood.

### 1.1. Fragile X Syndrome

Fragile X syndrome is the leading known inherited cause of intellectual disability (ID) [5] and presents with a variable clinical phenotype that is characterized by mild to severe ID, anxiety, social deficits, abnormalities in communication, gaze aversion, inattention, hyperactivity, impulsivity, and aggression [6,7,8]. FXS is associated with a mutation on an unstable trinucleotide (CGG) repeat expansion on the fragile X mental retardation 1 gene (FMR1). Prevalence rates for FXS are known to vary; however, it is estimated to occur in approximately 1 in 5000 to 7000 males and 1 in 4000 to 11000 females [9,10]. Since the *FMR1* gene is on the X chromosome, this results in the majority of males with FXS experiencing a more severe clinical presentation in comparison to females with FXS. Thus, the literature has focused primarily on males with FXS with the phenotypic profile for males with FXS being well established. Specifically, males with FXS often present with moderate to severe ID, comorbid ASD, self-injurious and aggressive behaviors, and attention deficits [6]. In contrast, the phenotypic profile for females with FXS is less predictable with more variability, including moderate to average or above average IQ with high rates of anxiety, depression, and attention problems [6]. However, the behavioral phenotype of females with FXS and how it compares to males with FXS remains unclear. 

Deficits in socialization in FXS are commonly associated with the presence of ASD symptoms. FXS is the most known monogenetic cause of ASD accounting for approximately 2% of all ASD cases [11], with comorbidity rates ranging from 50–70% in males, 16–20% in females, and 30–60% in all children with FXS [12,13,14]. There remains a controversy in the literature about the significant overlap of ASD symptoms in FXS. Some suggest that FXS be considered a subtype of the autism spectrum, whereas others suggest they are two distinct conditions with fundamental differences [12,15,16,17]. Overall, individuals with FXS who meet diagnostic criteria for ASD are at risk for a higher prevalence of behavioral problems and significantly poorer outcomes in comparison to individuals with only FXS [12,15]. Further, controversy has also emerged about the impact of intellectual impairments and whether low IQ is driving the ASD behavioral phenotype found in FXS rather than the presence of ASD itself. Across a variety of genetic conditions at higher risk for ASD, the literature has supported that groups with higher levels of IQ are at substantially reduced risk of ASD symptomology compared to groups with lower IQ [18]. Given intellectual impairments are a core feature of the FXS phenotype, it is important to assess for problems that exist or remain above and beyond the impact of intellectual functioning, especially when trying to understand the impact of ASD symptoms.

### 1.2. Restricted Repetitive Behaviors

A unique facet of RRBs is their occurrence in normal development. Typically developing children often engage in a variety of repetitive motor (e.g., flapping, rocking) or compulsive and ritualistic behaviors (e.g., daily routines/rituals). They often appear in the first year of life, increase up through age three, and decline after age 4 [19]. For example, Thelen [20] reported that typically developing infants engage in a series of rhythmic, repetitive behaviors such as kicking, banging, rocking, waving, and mouthing that often peak in frequency around 24 months of age. More complex repetitive behaviors in the form of ritualized behaviors begin to emerge as early as the second year of life where children begin to show rigidity or inflexibility [19]. Evans et al. [19] found that 75% of their sample aged 24 to 47 months engage in compulsive-like behaviors (e.g., being perfectionistic, having bedtime routines, having preferences for certain foods or clothes). Young children may also show compulsive ordering and arranging until some subjective sensory-perceptual criteria of “just right” is met [19]. Further, attachment to certain objects and intense, restricted interests are also common in young children. For example, DeLoache et al. [21] found intense interests emerge in typically developing children during the first two years of life, with some children developing odd interests (e.g., blenders, vacuum cleaners, fans, injuries) that preoccupied them for anywhere from months to years, and were much more common in boys than girls. Importantly, the occurrence of RRBs has been theorized to have important adaptive functions during early development [20]; however, the point at which these behaviors transition from normative to pathological remains blurred.

Aside from RRBs occurring in typical development, they also co-occur in a variety of developmental disabilities, psychiatric conditions (e.g., schizophrenia, obsessive-compulsive disorder) and neurological disorders (e.g., Tourette syndrome, Parkinson’s disease, Alzheimer’s). The presence of RRBs are more commonly noted in ASD as this is a core area of impairment. Within ASD, RRB’s occur more frequently than typically developing individuals, persist across time, and impact appropriate cognitive and social development [22,23,24]. The commonality of RRBs across a wide variety of disorders poses the question as to whether there are distinct RRB profiles within these clinical populations or is there some shared underlying pathophysiological mechanism across disorders.

#### Restricted Repetitive Behaviors in FXS

The FXS phenotype is associated with a generally increased risk for RRBs, including verbal perseveration, hand flapping, body rocking, compulsive behaviors, and self-injurious behaviors (SIB) [5,25,26,27]. These behaviors have also been targeted in a number of FXS clinical trials given their high prevalence and severity [28]. Only recently has the research begun to explore the RRB profile in FXS and how the different subcomponents of RRBs map onto the FXS phenotype. In comparison to other genetic syndromes, individuals with FXS across a wide age range exhibit higher rates of hand stereotypies, lining up objects, restricted conversations, preference for routines, and echolalia [27]. Further, “just right” behaviors (e.g., insistence on objects, furniture, toys remaining in the same place) were found to correlate with autism symptoms as measured by an ASD questionnaire. In a group of young boys with FXS and ASD, Wolff et al. [3] found a significant overlap in stereotypy, sameness behaviors, and SIB in comparison to young boys with ASD only, but significantly less compulsive or ritualistic behaviors in the boys with FXS and ASD. These findings suggest that lower rates of more complex forms of RRBs (e.g., compulsive and ritualistic behaviors) may be specific to the FXS behavioral profile; however, individuals with FXS and ASD closely resemble those with idiopathic ASD.

One study has sought to understand the RRB profile specific to boys with FXS ages 6 to 10 years [2]. Oakes et al. [2] found that Ritualistic/Sameness and Sensory-Motor behaviors were the most frequently endorsed problem behaviors by their caregivers, with SIB being the least problematic concern. Combining these results with Wolff et al. [3], these findings suggest the RRB profile in FXS is relatively stable across preschool years through middle childhood. Further, Oakes et al. [29] found at the item level that caregivers endorsed hand/finger mannerisms, sensory behaviors, preoccupations with objects or activities, strong attachment to one object, difficulty with transitions, sleep/bedtime rituals, completeness (e.g., doors must be open or closed), and not wanting to visit new places as the most severe problems. Lastly, higher rates of restricted interests were significantly related to lower nonverbal IQ and increased social affective deficits specific to ASD. Unfortunately, little is known about the RRB profile in adolescents and adults with FXS. Recently, Moskowitz et al. [4] utilized longitudinal models to examine RRBs through adolescence in males and females with FXS. Results suggested the developmental stability of more complex forms of RRBs (Restricted Interests, Compulsive, and Ritualistic/Sameness behaviors) aligning with previous findings suggesting individuals with FXS exhibit a minimal change in RRBs across time [30] and chronological age [2,3,31].

In addition to the limited literature across the lifespan, there is also limited information about the specific RRB profile in females with FXS. Moskowitz et al. [4] found differential trends of RRB profiles based on gender. Specifically, they found males with FXS exhibit higher rates of Sensory-Motor, Restricted Interests, SIB, and Ritualistic/Sameness behaviors across age with no difference in Compulsive behaviors between males and females. Further, Hall et al. [26] found that females with FXS exhibited lower rates of SIB and compulsive behaviors in comparison to males with FXS. Otherwise, the prevalence and severity of RRBs across multiple subtypes remain unclear in females with FXS after the age of 18. Characterizing the prevalence and severity of RRBs in males and females with FXS are important for delineating distinct behavioral phenotypes and their role in development. Further, understanding the neurobiological underpinnings of RRBs in FXS can help inform targeted behavioral and pharmacological treatment. 

### 1.3. The Present Study

The present study aims to further the field’s understanding of the RRB profile across a wide age range of males and females with FXS through a commonly used caregiver report measure of RRBs, the Repetitive Behavior Scale-Revised (RBS-R) [24]. To do so, the present study examines item level and subscale level differences across age, gender, and ASD diagnostic status. It is hypothesized that RRBs will remain relatively stable across age. It is also hypothesized that males with FXS will exhibit higher rates of RRBs across the item and subscale level. Lastly, it is hypothesized that individuals with FXS and a diagnosis of ASD will also exhibit higher levels of RRBs in the areas of Sensory Motor, Ritualistic/Sameness, Restricted Interests, and Compulsive Behaviors. Given low intellectual functioning is a core feature of FXS and is difficult to separate from the common ASD symptoms reported, the present study also explores the role of gender and ASD diagnostic status while controlling for IQ to see what differences remain above and beyond intellectual functioning. Lastly, the present study explores the relationships between RBS-R subscale scores and clinical measures specific to other problem behaviors and ASD symptoms. 

## 2. Materials and Methods

### 2.1. Participants

Data analyzed for this study were derived from an extension of the Fragile X Online Registry With Accessible Research Database (FORWARD) using a multisite design (Rush University Medical Center, Chicago; Cincinnati Children’s Hospital Medical Center; Children’s Hospital Colorado/University of Colorado, Denver) to collect additional essential longitudinal phenotyping data in individuals with FXS through a comprehensive core battery of outcome measures administered yearly. The FORWARD database is one of the largest resources of clinical and demographic data for individuals with FXS in the United States [32]. Data for this study included baseline data from FORWARD—Component C for 154 males (*N* = 112) and females (*N* = 42) with FXS evaluated between 2015 and 2019, who had data available for the measures of interest. Given these data are a part of the longitudinal database, information is only available for individuals with a diagnosis of FXS and there were no data available on typically developing individuals. Participants were between the age of 2 and 50 (*M* = 16.64, SD = 11.35) with a confirmed molecular diagnosis of FXS. All participants or their guardians provided written informed consent and participant assent (if feasible) for study participation. The study was approved by the Institutional Review Board (IRB) at all three participating centers: Cincinnati Children’s Hospital Medical Center Institutional Review Board (IRB#: 2012-2445); Colorado Multiple Institutional Review Board (IRB#: 15-1538); Rush University Office of Research Affairs (IRB#: 08121202).

### 2.2. Measures

Demographic data, including age, gender, and race/ethnicity, were collected using a clinician report form. The clinician report form also collected information on participants’ current ASD diagnostic status (39% of our sample had a diagnosis of ASD; 43% of males and 28% of females). Participants’ cognitive functioning was assessed using the Stanford Binet Intelligence Scales-Fifth Edition (SB-5) [33]. Given that the majority of the participants obtained a full-scale IQ score at or close to the floor score (40) of the test (males: 45% with a score of 40 and 81% ≤50; females: 13% with a score of 40 and 39% ≤50), deviation scores were calculated and used in all the statistical models for verbal and nonverbal IQ using the technique proposed by Sansone and colleagues [34]. Participants’ caregivers also completed the Aberrant Behavior Checklist (ABC) [35], Social Communication Questionnaire (SCQ) [36], Social Responsiveness Scale-Second Edition (SRS-2) [37], and the Vineland Adaptive Behavior Scales-Third Edition (Vineland-3). The ABC is a caregiver-informed problem behavior scale that assesses five categories: Irritability, Lethargy/Social Withdrawal, Stereotypic Behavior, Hyperactivity, and Inappropriate Speech. Factor analysis of the ABC supported a six-factor structure for use in FXS [38]. Therefore, the six-factor model was used in the present study which includes an additional sixth factor, Social Avoidance. The SCQ is a caregiver-informed behavioral checklist on certain social behaviors, communication behaviors, abnormal language use, and stereotyped behaviors that map on common symptoms found in ASD. Cut-off scores of greater than 15 on the SCQ are indicative of possible ASD. The SRS-2 is also a caregiver informed measure of symptoms associated with autism that assesses five categories: Social Awareness, Social Cognition, Social Communication, Social Motivation, and Restricted Interests/Behaviors. The Vineland-3—Interview Form was administered to all caregivers to assess current adaptive behavior functioning across areas of communication, daily living skills, social skills, and motor skills and provides an overall Adaptive Behavior Composite score. See Table 1 for participant descriptive statistics along with the caregiver rating scales for the analyzed sample.

Participants’ caregivers completed the Repetitive Behavior Scale-Revised (RBS-R) [24]. The RBS-R is comprised of 43 items that measure RRBs and is normed on individuals with ID. Items are rated on a 4-point Likert scale: 0 = behavior does not occur; 1 = behavior occurs and is a mild problem; 2 = behavior occurs and is a moderate problem; 3 = behavior occurs and is a severe problem. Items are grouped into six subscales: Stereotyped Behavior; Self-injurious Behavior; Compulsive Behavior; Ritualistic Behavior; Sameness Behavior; and Restricted Behavior. Two scores can be derived from the subscales, one based on the summed scores for each subscale, and one based on the number of items endorsed for each subscale. A total score can also be calculated by summing the scores or summing the number of items endorsed across all items, although not recommended for use [39,40]. There have been several empirical evaluations of the factor structure of the RBS-R in the literature [40,41,42,43]. A recent psychometric analysis across a variety of proposed models for interpreting the RBS-R provided continued evidence to support the use of a five-factor model. Hooker et al. [43] reported higher reliability across factors, with little difference between extracted scores and summed scores, and small, significant associations with diagnostic measures using the five-factor approach. Therefore, the present study chose to use the five-factor model identified by Bishop and colleagues [42], which is consistent with previous work examining RRBs in young boys with FXS [2].

### 2.3. Procedures

Participants were assessed in the clinic by trained examiners administering the SB-5 and the participant’s caregivers completed the RBS-R, SCQ, SRS-2, and ABC. Trained examiners administered the Vineland-3 to the participant’s caregivers. The SB-5 was not able to be completed on all participants due to behavioral concerns or functioning level. Some of the participants did not have SRS-2, SCQ, ABC, or Vineland-3 data due to data collection errors (e.g., caregivers forgot to fill out the form during the visit, tried to obtain through mail and caregivers never mailed back forms). Further, the SCQ and SRS were only administered to children 4 years or older. 

### 2.4. Data Analysis

Preliminary analyses were conducted to examine outliers, non-normality, linearity, and homogeneity of residuals. There were no significant cross-site differences observed for the primary measure of interest (*ps* > 0.63). First, Spearman correlations were utilized to assess the relationship between age, IQ, and clinical measures across the RBS-R subscales. Next, descriptive examinations of caregiver ratings at the item level were assessed by graphing the percentage of caregivers who endorsed the presence of individual behaviors as being moderate to severe problems within the total sample and based on gender and ASD diagnosis. Additionally, descriptive examinations of caregiver ratings at the subscale level were assessed by averaging the item level ratings of moderate to severe problems within each subscale across age, gender, and ASD diagnosis. Independent sample t-tests were also utilized to examine differences between gender and ASD diagnosis at the item level and subscale level. Levene’s Test for Equality of Variances was assessed for each model with equal variances not assumed *t*-values and *p*-values reported when necessary. Lastly, MANCOVA models were utilized to examine the effects of gender and ASD diagnosis on the RBS-R subscales while controlling for verbal IQ, nonverbal IQ, and age. 

## 3. Results

### 3.1. Correlations with Clinical Measures

Spearman correlations were used to examine the extent to which age, verbal IQ, nonverbal IQ, the Vineland ABC, the ABC subscales, the SRS-2 restricted and repetitive behaviors t-score, the SRS-2 total t-score, and the SCQ total score were predictively correlated with the RBS-R subscales (Table 2). Age was significantly negatively correlated with the Sensory-Motor (*p* = 0.001) subscale. Nonverbal IQ was significantly negatively correlated with all of the subscales except for Compulsive/Ritualistic (*p* = 0.102). Verbal IQ was significantly negatively correlated with all of the RBS-R subscales (*ps* < 0.033). The Vineland-3 Adaptive Behavior Composite was significantly negatively correlated with all of the subscales except for Compulsive/Ritualistic (*p* = 0.265). The ABC Social Avoidance subscale was significantly positively correlated with all of the subscales except for Sensory-Motor (*p* = 0.093). There were significant positive correlations for all of the RBS-R subscales the other ABC subscales, SRS-2 RRB, SRS-2 Total T-Score, and the SCQ (*ps* < 0.002). The most significant correlations (*r* > 0.5) were found in the relationship between (1) the RBS-R Sensory-Motor subscale and ABC Stereotypy, ABC Hyperactivity, SRS-2 Total, SRS-2 RRB, and SCQ total scales, (2) the RBS-R Restricted Interests and SRS-2 RRB, SRS-2 Total scales, and (3) RBS-R Ritualistic/Sameness and ABC Irritability, SRS-2 RRB, and SRS-2 Total domains.

### 3.2. Item Level Differences

**Figure 1:**
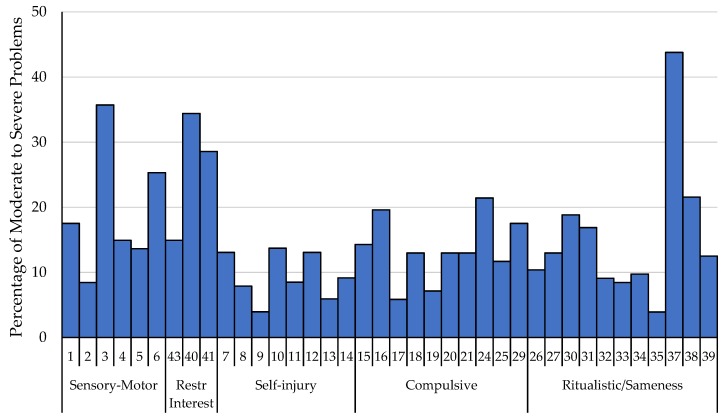
Average percentage of caregivers reporting behaviors to be a moderate to severe problem (a score of 2 or 3) for the total sample across the RBS-R items.

Participants’ mean item-level ratings for the entire sample were assessed (Table 3). The five highest-rated items for the entire sample included (1) resisting change in activities/difficulties with transitions, (2) hand/finger repetitive behaviors, (3) fascination or preoccupation with one subject or activity, (4) strongly attached to one specific object, and (5) sensory repetitive behaviors. The percentage of caregivers reporting behaviors in the moderate to severe problem range by item are represented in Figure 1. 

#### 3.2.1. Gender

The five highest-rated items for males with FXS included: (1) resisting change in activities/difficulties with transitions, (2) hand/finger repetitive mannerisms, (3) fascination or preoccupation with one subject or activity, (4) strongly attached to one specific object, and (5) sensory repetitive behaviors. The five highest-rated items for females with FXS included: (1) resisting change in activities/difficulties with transitions, (2) fascination or preoccupation with one subject or activity, (3) becomes upset if interrupted in what he/she is doing, (4) strongly attached to one specific object, and (5) hoarding/saving items. 

**Figure 2:**
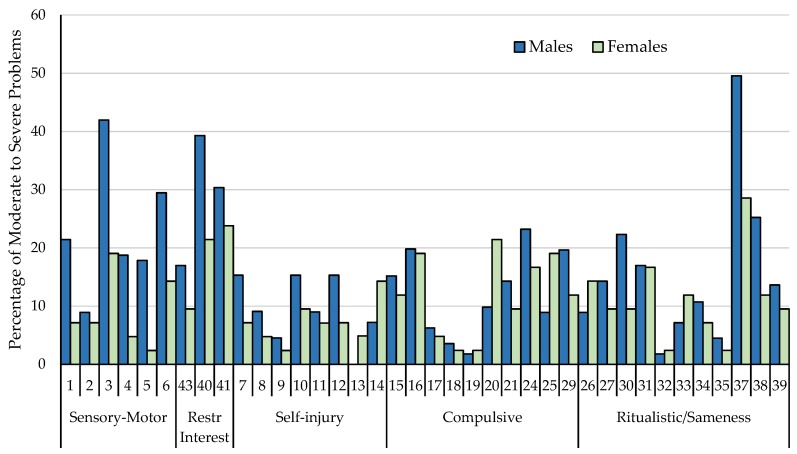
The average percentage of caregivers reporting behaviors to be a moderate to severe problem (a score of 2 or 3) between males and females with fragile X syndrome (FXS) across the RBS-R items.

Independent samples t-tests were used to assess differences at the item level based on gender. Results suggest significant differences primarily emerge in the Sensory-Motor subscale with males having higher scores on the whole body (*t* = 2.65, *p* = 0.009), hand/finger (*t* = 3.42, *p* = 0.001), locomotion (*t* = 3.79, *p* = 0.000), object usage (*t* = 5.30, *p* = 0.000), and fascination/preoccupation with movement (*t* = 2.79, *p* = 0.006) items in comparison to females. Additionally, males with FXS were rated significantly higher in their fascination/preoccupation with one subject or activity (*t* = 2.41, *p* = 0.018), disliking changes in the appearance or behavior of other people around him/her (*t* = 2.04, *p* = 0.044), and resisting changes in activities/difficulty with transitions (*t* = 2.72, *p* = 0.008). Overall, females were rated lower than males across the majority of the RBS-R items (See Table 3). The percentage of caregivers reporting behaviors in the moderate to severe problem range by item and gender are represented in Figure 2.

#### 3.2.2. ASD Diagnosis

The five highest rated items for individuals with FXS and an ASD diagnosis included: (1) resisting change in activities/difficulties with transitions, (2) hand/finger mannerisms, (3) fascination or preoccupation with one subject or activity, (4) strongly attached to one specific object, and (5) sensory repetitive behaviors. The five highest rated items for individuals with FXS without an ASD diagnosis included: (1) resisting change in activities/difficulties with transitions, (2) hand/finger mannerisms, (3) fascination or preoccupation with one subject or activity, (4) sensory repetitive behaviors, and (5) becomes upset if interrupted in what he/she is doing. 

**Figure 3:**
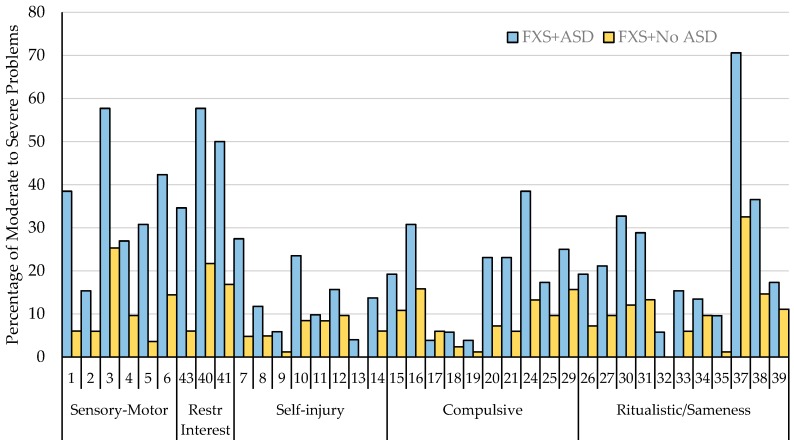
The average percentage of caregivers reporting behaviors to be a moderate to severe problem (a score of 2 or 3) between individuals with FXS and a comorbid diagnosis of ASD and those without a comorbid diagnosis of ASD across the RBS-R items.

Independent samples t-tests were used to assess differences at the item level based on ASD diagnosis. For all of the items on the Sensory-Motor and Restricted Interest subscales of the RBS-R, individuals with FXS and ASD had significantly higher scores compared to those with FXS without ASD (*ps* < 0.002). Additional significant differences for the other domains of the RBS-R are reported in Table 3. The most significant differences (>0.50 difference) between individuals with FXS and ASD and those without ASD were found for the following items: (1) strongly attached to one specific object, (2) fascination/preoccupation with movement, (3) whole body, (4) fascination/preoccupation with one subject or activity, (5) hand/finger, (6) resists changing activities/difficulty with transitions, (7) sensory, (8) object usage, (9) hits self with body part, (10) sleeping/bedtime, (11) locomotion, (12) and objects to visiting new places. Overall, individuals with FXS and a diagnosis of ASD have higher mean scores across all of the RBS-R items in comparison to those without an ASD diagnosis (See Table 3). The percentage of caregivers reporting behaviors in the moderate to severe problem range by item and ASD diagnosis are represented in Figure 3.

### 3.3. Subscale Level Differences

Participants’ subscale level responses were calculated by summing item scores within each factor and taking the mean. Utilizing the percentage of moderate to severe problems reported by caregivers at the item level, average percentages of moderate to severe problems were also calculated for the RBS-R subscales. Participant’s caregivers reported Restricted Interests (31.5%), Sensory-Motor (18%), and Ritualistic/Sameness (15.3%) behaviors as most problematic in comparison to Compulsive (13.6%) and Self-injurious (9.4%) behaviors. 

#### 3.3.1. Age

**Figure 4:**
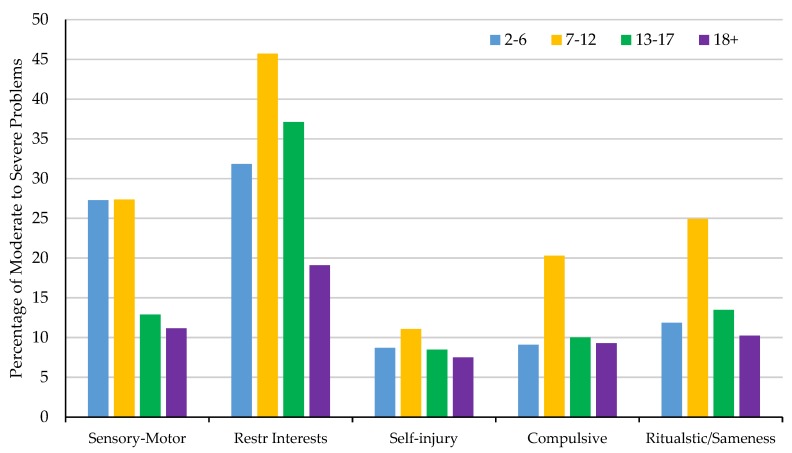
The average percentage of caregivers reporting behaviors to be a moderate to severe problem (a score of 2 or 3) between 2–6, 7–12, 13–17, and 18+ years of age for the RBS-R subscales.

Utilizing the percentage of moderate to severe problems reported by caregivers at the item level, average percentages for moderate to severe problems were calculated for the RBS-R subscales across 4 age groups: 2–6 years, 7–12 years, 13–17 years, and 18+ years (Figure 4). Overall, Restricted Interests, Sensory-Motor, and Ritualistic/Sameness behaviors were the most reported problems across all age groups. Further, severity of RRBs appear to peak between ages 7 to 12 in FXS and then decline across age for the majority of the RRB subscales with the exception of Sensory-Motor behaviors. Sensory-Motor behaviors appear to peak in severity and remain stable between 2 and 12 years of age before declining. 

#### 3.3.2. Gender

Utilizing the percentage of moderate to severe problems reported by caregivers at the item level, average percentages of moderate to severe problems were calculated for the RBS-R subscales by gender. For males with FXS, caregivers reported Restricted Interests (34.8%), Sensory-Motor (22.2%), and Ritualistic/Sameness (15.9%) behaviors as most problematic in comparison to Compulsive (12.2%) and Self-injurious (9.5%) behaviors. For females with FXS, caregivers reported Restricted Interests (22.6%), Compulsive (11.9%), and Ritualistic/Sameness (11.3%) behaviors as most problematic in comparison to Sensory-Motor (9.2%) and Self-injurious (7.2%) behaviors. Within the RBS-R subscales, the Sensory-Motor subscale emerged as the only subscale with significant differences between males and females (*t* = 4.03, *p* = 0.000). Specifically, males with FXS (*M* = 5.37, SD = 4.30) were rated higher than females with FXS (*M* = 2.57, SD = 3.64). See Table 4. 

#### 3.3.3. ASD Diagnosis

Utilizing the percentage of moderate to severe problems reported by caregivers at the item level, average percentages of moderate to severe problems were calculated for the RBS-R subscales by ASD diagnosis. For individuals with FXS and a diagnosis of ASD, caregivers reported Restricted Interests (53.8%), Sensory-Motor (35.2%), and Ritualistic/Sameness (24.6%) behaviors as most problematic in comparison to Compulsive (19%) and Self-injurious (14%) behaviors. For individuals with FXS without a diagnosis of ASD, caregivers reported Restricted Interests (19.3%), Ritualistic/Sameness (10.7%), and Sensory-Motor (10.1%) behaviors as most problematic in comparison to Compulsive (8.8%) and Self-injurious (5.4%) behaviors. Within the RBS-R subscales, all of the subscales were significantly different between those with and without an ASD diagnosis. Within the Sensory-Motor (*t* = 6.01, *p* = 0.000), Restricted Interests (*t* = 5.04, *p* = 0.000), Self-injury (*t* = 3.36, *p* = 0.001), Compulsive (*t* = 2.66, *p* = 0.010), and Ritualistic/Sameness (*t* = 3.86, *p* = 0.000) subscales, individuals with FXS and a diagnosis of ASD have significantly higher scores than those without a diagnosis with ASD. See Table 4.

### 3.4. Impact of Gender and ASD Diagnosis on RBS-R Scores

A series of MANCOVA’s were used to examine the effects of gender and ASD diagnosis on the RBS-R subscales while controlling for verbal IQ, nonverbal IQ, and age. 

#### 3.4.1. Sensory-Motor

For the Sensory-Motor subscale, there were no significant main effects of nonverbal IQ (*F*[1,99] = 0.44, *p* = 0.509, partial *η^2^* = 0.01). Significant main effects were found for verbal IQ (*F*[1,99] = 36.28, *p* = 0.000, partial *η^2^* = 0.27), age (*F*[1,99] = 12.60, *p* = 0.001, partial *η^2^* = 0.11), gender (*F*[1,99] = 4.20, *p* = 0.043, partial *η^2^* = 0.04) and ASD diagnosis (*F*[1,99] = 14.00, *p* = 0.000, partial *η^2^* = 0.12). A significant interaction was found between gender and ASD diagnosis (*F*[1,99] = 4.99, *p* = 0.028, partial *η^2^* = 0.05). Tukey’s HSD posthoc comparisons revealed males with FXS and diagnosis of ASD (*M* = 6.75, SE = 0.65) exhibit higher rates of Sensory-Motor behaviors in comparison to males with FXS without a diagnosis of ASD (*M* = 3.29, SE = 0.46); however, this difference did not emerge between females with FXS who have (*M* = 2.68, SE = 1.16) and who do not have an ASD (*M* = 2.54, SE = 0.69) diagnosis (*t* = 4.34, *p* = 0.000).Further, males with FXS and a diagnosis of ASD also exhibited higher rates of Sensory-Motor behaviors in comparison to females with FXS and a diagnosis of ASD (*t* = 4.01, *p* = 0.001).

#### 3.4.2. Restricted Interests

For the Restricted Interests subscale, there were no main effects of nonverbal IQ (*F*[1,99] = 2.22, *p* = 0.139, partial *η^2^* = 0.02) and gender (*F*[1,99] = 0.03, *p* = 0.857, partial *η^2^* = 0.00). Significant main effects emerged for verbal IQ (*F*[1,99] = 20.60, *p* = 0.000, partial *η^2^* = 0.17), ASD diagnosis (*F*[1,99] = 8.67, *p* = 0.004, partial *η^2^* = 0.08), and age(*F*[1,99] = 4.76, *p* = 0.032, partial *η^2^* = 0.05). Tukey’s HSD posthoc comparisons revealed individuals with FXS and a diagnosis of ASD (*M* = 2.50, SE = 0.36) had higher rates of Restricted Interest behaviors in comparison to those without ASD (*M* = 1.61, SE = 0.22; *t* = 2.01, *p* = 0.047). There was no significant interaction between ASD diagnosis and gender (*F*[1,99] = 0.01, *p* = 0.955, partial *η^2^* = 0.00).

#### 3.4.3. Self-Injury

For the Self-injury subscale, there were no significant main effects of nonverbal IQ (*F*[1,99] = 0.17, *p* = 0.673, partial *η^2^* = 0.00), gender (*F*[1,99] = 0.49, *p* = 0.482, partial *η^2^* = 0.01), ASD diagnosis (*F*[1,99] = 2.64, *p* = 0.106, partial *η^2^* = 0.03), and age (*F*[1,99] = 0.33, *p* = 0.564, partial *η^2^* = 0.00). Significant main effects emerged for verbal IQ (*F*[1,99] = 6.69, *p* = 0.011, partial *η^2^* = 0.06). There was no significant interaction between ASD diagnosis and gender (*F*[1,99] = 0.02, *p* = 0.887, partial *η^2^* = 0.00).

#### 3.4.4. Compulsive

For the Compulsive subscale, there were no significant main effects of nonverbal IQ (*F*[1,99] = 0.14, *p* = 0.708, partial *η^2^* = 0.01), gender (*F*[1,99] = 0. 52, *p* = 0. 471, partial *η^2^* = 0.01), ASD diagnosis (*F*[1,99] = 1.31, *p* = 0.253, partial *η^2^* = 0.01), and age (*F*[1,99] = 2.16, *p* = 0.144, partial *η^2^* = 0.02). Significant main effects emerged for verbal IQ (*F*[1,99] = 6.62, *p* = 0.011, partial *η^2^* = 0.06). There was no significant interaction between ASD diagnosis and gender (*F*[1,99] = 0.07, *p* = 0.788, partial *η^2^* = 0.00).

#### 3.4.5. Ritualistic/Sameness

For the Ritualistic/Sameness subscale, there were no significant main effects of nonverbal IQ (*F*[1,99] = 1.82, *p* = 0.179, partial *η^2^* = 0.02), gender (*F*[1,99] = 0.12, *p* = 0.721, partial *η^2^* = 0.00), ASD diagnosis (*F*[1,99] = 3.71, *p* = 0.056, partial *η^2^* = 0.04), and age (*F*[1,99] = 2.81, *p* = 0.096, partial *η^2^* = 0.03). Significant main effects emerged for verbal IQ (*F*[1,99] = 12.03, *p* = 0.001 partial *η^2^* = 0.11). There was no significant interaction between ASD diagnosis and gender (*F*[1,99] = 0.15, *p* = 0.697, partial *η^2^* = 0.00).

## 4. Discussion

### 4.1. Summary of Findings

Although the presence of RRBs are core to the diagnosis of ASD, they are also quite common in typically developing children and have been found in a variety of other clinical and neurodevelopmental disorders, including FXS. At the global level, individuals with FXS are known for exhibiting RRBs, with a unique RRB profile being observed across the preschool years through adolescence [2,3,4], with developmental stability across time [30] and age [2,3,4,31]. To date, there has been little work examining the FXS RRB profile across the lifespan through adulthood, with limited information about the female RRB profile [4,26]. Further, the FXS RRB profile is suggested to resemble idiopathic ASD across several sublevels of RRBs (e.g., stereotypy, sameness, and SIB); however, rates of Compulsive, Sensory-Motor, and Ritualistic behaviors may also be specific to the FXS profile [2,27]. The present study aimed to build on the fields current understanding of the FXS RRB phenotypic profile up through adulthood, while teasing apart differences between males and females with FXS and expanding on the impact of a comorbid ASD diagnosis on RRB severity. This study is one of the first to provide detailed findings about differences in RRB profiles at the item and subtype level based on gender.

Overall, the present study builds on the premise that higher ratings of RRBs are associated with poorer outcomes in FXS [12,15]. Specifically, lower IQ, lower adaptive behavior skills, and higher rates of problem behavior and ASD symptomology were related to higher rates of RRBs. Although age was not significantly correlated with the majority of the RBS-R subscales, severity of problems reported by caregivers suggest the FXS RRB profile differs based on age. Specifically, severity of more complex forms of RRBs (Restricted Interests, Compulsive, and Ritualistic/Sameness behaviors) appear to peak between 7 and 12 years of age before declining and becoming stable across adolescence and adulthood. Sensory-Motor behaviors appear to peak even earlier in childhood between 2 and 6 years, remain stable across age 12, and then decline and become stable across adolescence and adulthood. Similar to Oakes et al. [2], SIBs were rated least problematic with these findings extending beyond middle childhood with the severity of SIBs remaining stable throughout the lifespan. Overall, RRB’s appear to decline across age in severity across all of the RRB subscales, which is inconsistent with previous literature suggesting stability up through adolescence [2,3,4]. These inconsistent findings could be due to the observed peak in severity across several of the RBS-R subscales for children between 7 and 12 years of age. Otherwise, the severity of RRBs outside of 7 to 12 years of age as rated by their caregivers aligns with previous literature suggesting stability across the lifespan with the exception of Sensory Motor behaviors.

Consistent with previous literature [2,3,4,27], the present study found certain sublevels of RRBs to be more problematic in FXS, building on the premise of a distinct RRB in profile. However, in contrast to Wolff et al. [3] and Oakes et al. [2], our findings revealed Restricted Interest behaviors to be most problematic for FXS caregivers, proceeded by Sensory-Motor and Ritualistic/Sameness behaviors; however, they did align with findings reported by Moskowitz et al. [4]. Given a different profile emerged in the present study, this suggests age may play a factor in these profiles given our age range extended beyond adolescence. Further, the present study also found Self-injurious behaviors to be least problematic [2]. When considering gender at the subscale level, some differences emerged as hypothesized but also some commonalities. Specifically, Restricted Interest behaviors were rated as most problematic in both males and females with FXS, with Ritualistic behaviors also being rated as third most problematic. However, males with FXS were rated as having more Sensory-Motor behaviors, whereas females with FXS were rated as having more Compulsive behaviors. Further, Sensory-Motor behaviors were the only area where both genders significantly differed in their severity ratings, suggesting males with FXS are more likely to engage in Sensory-Motor behaviors in comparison to females. Not only may there be a specific FXS RRB profile that potentially shifts with age, but there are also differences in profiles based on gender which helps lay important groundwork for targeted treatment development.

When considering the impact of ASD diagnosis on the RRB profile of FXS, the same core problems were reported in individuals with FXS and ASD (e.g., Restricted Interests, Sensory-Motor, and Ritualistic/Sameness behaviors being most problematic) as the overall sample; however, the severity of these problems almost doubles in comparison to individuals with FXS only, with significant differences emerging across all of the RBS-R subscales. These findings build on the premise that although these problems may be core to the FXS behavioral phenotype, the added diagnosis of ASD increases the risk for more problem behaviors in FXS including increased RRBs [12,15,30,31]. These findings suggest the RBS-R may be a valid measure for differentiating individuals with FXS who have ASD in comparison to those without ASD based on severity of ratings.

At the item level, some common problems emerged in FXS within the Sensory-Motor, Restricted Interests, and Ritualistic/Sameness subscales. Within the entire sample, FXS caregivers rated resisting changes in activities/difficulty with transitions and hand/finger mannerisms as the most problematic followed by fascination with one subject or activity, strongly attached to one specific object, and sensory repetitive behaviors. Interestingly, difficulty with transitions were most problematic for both males and females, supporting that this is core to the FXS behavioral phenotype. As hypothesized, some differences were identified based on gender. In comparison to males with FXS, caregivers of females with FXS endorsed becoming upset if interrupted in what he/she is doing and hoarding/saving items. Aligning with the subscale finding, hand/finger mannerisms and sensory behaviors appear to be specific problems in males with FXS. Overall, significant differences at the item level were found across several of the items on the RBS-R based on gender (Table 3), but not to the degree that was hypothesized. Females were primarily rated with less severe RRBs in comparison to males with FXS, which aligns with the fields current understanding about males with FXS being more severely impacted than females, but may not be to the same degree as other problem behaviors commonly found in FXS [6].

Similar to the subscale findings based on ASD diagnosis, the top-rated problems in those with FXS and diagnosis of ASD closely aligned to those with FXS only. Within the top five items endorsed based on ASD diagnosis, differences emerged where individuals with FXS and ASD were reported to have higher ratings of being strongly attached to one specific object, whereas individuals with FXS only were reported to have higher ratings of being upset if interrupted in what he/she is doing. Not only at the subscale level may the RBS-R be able to differentiate ASD diagnosis in FXS, but also the item level. Overall, individuals with FXS and ASD were rated as significantly higher for a large portion of items on the RBS-R; however, not to the degree that was hypothesized. It appears there are specific items within the RBS-R subscales that are driving the subscale differences that were found. These findings are important for understanding the utility of the RBS-R in FXS. Specifically, the RBS-R subscales may not be best interpreted at face value in FXS given item level analyses provide more nuanced details into understanding how ASD impacts specific RRBs found in FXS.

Given the controversy of IQ possibly driving the ASD symptoms seen in FXS and other genetic syndromes at high risk for ASD [18], the present study aimed to examine these differences further by assessing if these differences remain when controlling for IQ and age. The current study’s findings suggest that only Sensory-Motor behaviors remain as problematic in males with FXS and a diagnosis of ASD when considering IQ and age. These findings map onto those reported by Wolff et al. [3] and suggest that high rates of Sensory-Motor problems in FXS are most likely driven by ASD itself, rather than IQ. Further, ASD is most likely driving the increased rates of Restricted Interests seen in individuals with FXS and ASD regardless of gender.

### 4.2. Limitations

Although the present study utilized a robust sample of FXS participants, there are still several limitations to consider. Specifically, the majority of the sample consisted of males with FXS and given a focus in this study was on gender differences, additional work is needed with larger samples of females with FXS to confirm these findings. Further, ASD comparisons were based on methods of ASD diagnosis that varied between sites, with some relying on Clinical Best Estimates or previous community diagnostic evaluations. It would be important to replicate the findings in ASD using consistent gold standard measures for ASD diagnoses to determine who meets and does not meet ASD diagnostic criteria. Additionally, the present study did not use an idiopathic ASD comparison group; therefore, findings related to ASD diagnoses are may not be specific to FXS and may look different when compared to idiopathic ASD. Lastly, several of the participants were not able to complete an IQ measure, which may have impacted the present study’s findings related to IQ. The use of a developmental delay comparison group could have furthered our understanding of IQ on RRBs. Further, the utilization of a typically developing control group would allow for exploration of RRB severity in FXS outside normal development of RRBs, especially with the examination of mental ages given the cognitive deficits found in FXS. Lastly, the present study utilized cross-sectional data of RRBs and the use of longitudinal data would have allowed for further exploration of the changes in RRBs across development [4].

## 5. Conclusions and Future Directions

Taken together, these findings build on the fields current understanding of RRBs in FXS, with unique RRB profiles emerging based on age and gender with increased severity of RRBs based on ASD diagnosis. Further, subscale differences may not provide the best picture into the core areas of impairment within the FXS RRB behavioral phenotype. Future researchers and clinicians should aim to assess impairment at the item level to develop a better understanding of RRBs in FXS, especially when considering the gender of the child and ASD diagnosis to form an appropriate plan of treatment. Although FXS is the leading inherited cause of ID, ID does not exclusively account for the RRBs found in FXS when considering sensory motor behaviors and restricted interests. Importantly, comorbid ASD in males with FXS appears to be a significant factor in the severity of Sensory-Motor behaviors, whereas comorbid ASD regardless of gender in FXS is playing a significant role in the severity of Restricted Interests. Future work should continue to explore the impact of IQ and ASD diagnosis on the RRB profile in individuals with FXS. The groundwork is still being laid toward understanding the presence of RRB subtypes in females with FXS. Additional work is needed with larger samples to see if the female RRB profile findings are consistent.

Given that sublevels of RRBs were able to be detected in FXS and those with a diagnosis of ASD above and beyond IQ, these findings open the door to another area of interest for identifying common pathways to ASD through monogenetic syndromes. Further, common treatments for sensory motor behaviors and restricted interests in ASD may also show promise for individuals with FXS and ASD. However, additional work is needed to determine how RRBs in individuals with FXS and a diagnosis of ASD are similar to or different from idiopathic ASD [3]. Future work should also look at the impact of other high prevalence problem behaviors in FXS (e.g., ADHD, anxiety) on RRBs. For example, Oakes et al. [2] found several of the RBS-R subscales to be associated with increased anxiety. It is important to tease apart how other common comorbid conditions in FXS impact RRBs beyond ASD to better tailor treatment.

Further, additional work is needed on a longitudinal scale. Although age was not a significant factor for the majority of the subscales in this study, longitudinal analyses would provide better insight into the changes in RRBs across the lifespan [4]. Using these baseline data, this research group will analyze longitudinal aspects of RRBs in future publications. Not only is it important to understand changes in RRBs across time in FXS, it is also critical to assess how effective the RBS-R can detect change in FXS through test-retest reliability. There have been several psychometric analyses of the RBS-R in ASD [40,41,42,43]; however, to the authors knowledge, there has been no work to date assessing the test-retest reliability of the RBS-R. Of note, the RBS-R subscales were all significantly correlated with the RRB scale of the SRS-2, adding to the validity of the measure in a sample with FXS. This is important given the increased need and interest for identifying valid and reliable measures that accurately quantify the core phenotypic symptoms of FXS for clinical trials [28,44]. The RBS-R has been used in a few randomized ASD clinical trials as an outcome measure [39,45,46,47]. Unfortunately, these provided limited evidence to support the RBS-R can measure change with treatment; although, there is also limited support for any treatment for RRBs in ASD. If the RBS-R has been determined to reliably detect change, this could be extremely beneficial for clinical trials examining pharmacological and behavioral treatments for RRBs in FXS. Lastly, the results from this study could be utilized to design interventions for these specific RRB profiles in FXS and tailor pharmacological treatment.

## Figures and Tables

**Table 1:** Participant characteristics.

	Total Sample			Males with FXS			Females with FXS		
Variable	*n*	Mean	*SD*	*n*	Mean	*SD*	*n*	Mean	*SD*
Age (years)	154	16.64	11.25	112	16.69	11.68	42	16.51	10.55
Verbal IQ	122	54.22	16.36	85	48.89	11.34	38	66.00	19.51
Nonverbal IQ	122	52.42	15.53	85	47.79	12.55	38	63.05	16.64
Deviation Verbal IQ	119	52.02	24.34	83	44.48	21.31	36	69.43	22.07
Deviation Nonverbal IQ	119	50.42	22.86	83	43.35	20.59	36	66.72	19.41
Adaptive Behavior Composite	119	55.08	21.28	91	50.65	18.06	28	69.50	24.73
SCQ	136	13.24	6.95	98	14.35	6.37	38	10.37	7.64
SRS-2 Total T-Score	133	67.74	12.70	98	68.05	11.86	35	66.89	14.99
ABC Irritability	145	13.20	11.62	106	14.01	11.26	39	11.00	12.45
ABC Lethargy	145	5.51	5.40	106	5.33	4.96	39	6.00	6.509
ABC Stereotypy	145	4.75	4.58	106	5.16	4.59	39	3.64	4.44
ABC Hyperactivity	145	9.58	7.48	106	10.49	7.49	39	7.10	6.97
ABC Social Avoidance	145	2.73	3.40	106	2.41	3.10	39	3.62	4.03
ABC Inappropriate Speech	145	3.87	3.18	106	4.19	3.12	39	3.00	3.26

*Note.* ABC = Aberrant Behavior Checklist; SCQ = Social Communication Questionnaire; SRS-2 = Social Responsiveness Scale—Second Edition; IQ = intelligence quotient.

**Table 2:** Correlations between the RBS-R subscales according to Bishop et al.’s (2013) five-factor structure and clinical measures.

Subscale	Age (*N* = 154)	Nonverbal IQ (*N* = 119)	Verbal IQ (*N* = 119)	Vineland ABC (*N* = 119)	ABC Social Avoidance (*N* = 145)	ABC Hyperactivity (*N* = 145)	ABC Inappropriate Speech (*N* = 145)	ABC Irritability (*N* = 145)	ABC Lethargy (*N* = 145)	ABC Stereotypy (*N* = 145)	SRS-2 RRB (*N* = 133)	SRS-2 Total (*N* = 133	SCQ Total (*N* = 136)
**Sensory-Motor**	−0.274 *	−0.423 *	−0.447 *	−0.272 *	0.140	0.538 *	0.258 *	0.486 *	0.357 *	0.746 *	0.494 *	0.514 *	0.600 *
**Restricted Interests**	−0.154	−0.372 *	−0.383 *	−0.231 *	0.299 *	0.390 *	0.320 *	0.454 *	0.307 *	0.440 *	0.565 *	0.567 *	0.477 *
**Self-injury**	−0.053	−0.274 *	−0.231 *	−0.199 *	0.353 *	0.302 *	0.283 *	0.465 *	0.259 *	0.263 *	0.384 *	0.429 *	0.339 *
**Compulsive**	−0.045	−0.151	−0.196 *	−0.103	0.336 *	0.360 *	0.391 *	0.457 *	0.323 *	0.254 *	0.476 *	0.454 *	0.341 *
**Ritualistic/Sameness**	−0.056	−0.328 *	−0.310 *	−0.287 *	0.380 *	0.497 *	0.443 *	0.580 *	0.434 *	0.398 *	0.617 *	0.565 *	0.464 *

*Note.* * = *p* < 0.05, IQ = intelligence quotient, ABC = Aberrant Behavior Checklist, SRS-2 = Social Responsiveness Scale-Second Edition, SCQ = Social Communication Questionnaire.

**Table 3:** Repetitive Behavior Scale-Revised (RBS-R) item means by overall sample, gender, and autism spectrum disorder (ASD) diagnostic status according to Bishop et al.’s (2013) 5-factor structure.

RBS-R Subscales by Bishop et al. (2013)	Item	Mean Score Total Sample (SD) (*N* = 154)	Mean Score Males (SD) (*N* = 112)	Mean Score Females (SD) (*N* = 42)	Mean Differences between Males and Females	Mean Score ASD Diagnosis (SD) (*N* = 52)	Mean Score No ASD Diagnosis (SD) (*N* = 83)	Mean Differences between ASD Diagnosis
**Sensory-Motor**	1. Whole Body	0.58 (0.91)	0.68 (0.97)	0.31 (0.68)	0.009 *^,†^	1.06 (1.13)	0.30 (0.62)	0.000 *^,†^
	2. Head	0.32 (0.66)	0.35 (0.69)	0.26 (0.59)	0.475	0.60 (0.85)	0.20 (0.54)	0.004 *^,†^
	3. Hand/finger	1.12 (1.03)	1.29 (0.99)	0.67 (1.00)	0.001 *	1.62 (1.05)	0.89 (0.92)	0.000 *
	4. Locomotion	0.54 (0.87)	0.67 (0.92)	0.19 (0.59)	0.000 *^,†^	0.88 (1.02)	0.36 (0.73)	0.002 *^,†^
	5. Object usage	0.58 (0.84)	0.74 (0.90)	0.17 (0.44)	0.000 *^,†^	0.96 (1.07)	0.36 (0.55)	0.000 *^,†^
	6. Sensory	0.89 (0.97)	0.96 (0.99)	0.69 (0.90)	0.121	1.35 (1.15)	0.64 (0.76)	0.000 *^,†^
	43. Fascination, preoccupation with movement	0.57 (0.90)	0.68 (0.94)	0.29 (0.71)	0.006 *^,†^	1.10 (1.14)	0.31 (0.62)	0.000 *^,†^
**Restricted Interests**	40. Fascination, preoccupation with one subject or activity	1.12 (1.00)	1.24 (0.98)	0.81 (0.99)	0.016 *	1.60 (1.03)	0.86 (0.90)	0.000 *
	41. Strongly attached to one specific object	0.92 (1.10)	0.97 (1.10)	0.76 (1.08)	0.285	1.46 (1.13)	0.61 (0.96)	0.000 *^,†^
**Self-injury**	7. Hits self with body part	0.44 (0.77)	0.50 (0.82)	0.29 (0.60)	0.072 ^†^	0.78 (0.90)	0.25 (0.54)	0.000 *^,†^
	8. Hits self against surface or object	0.32 (0.70)	0.36 (0.75)	0.19 (0.51)	0.105 ^†^	0.47 (0.76)	0.21 (0.56)	0.035 *^,†^
	9. Hits self with object	0.20 (0.54)	0.23 (0.59)	0.12 (0.40)	0.193 ^†^	0.27 (0.56)	0.11 (0.35)	0.067 ^†^
	10. Bites self	0.51 (0.86)	0.57 (0.88)	0.36 (0.79)	0.177	0.78 (1.06)	0.34 (0.67)	0.009 *^,†^
	11. Pulls hair or skin	0.31 (0.68)	0.32 (0.71)	0.31 (0.60)	0.963	0.37 (0.77)	0.29 (0.62)	0.492
	12. Rubs or scratches self	0.45 (0.78)	0.48 (0.82)	0.38 (0.70)	0.500	0.47 (0.86)	0.42 (0.70)	0.719
	13. Inserts finger or object	0.07 (0.31)	0.05 (0.21)	0.15 (0.48)	0.196 ^†^	0.14 (0.45)	0.04 (0.19)	0.067 ^†^
	14. Skin picking	0.36 (0.74)	0.30 (0.71)	0.52 (0.80)	0.113 ^†^	0.45 (0.88)	0.27 (0.61)	0.188 ^†^
**Compulsive**	15. Arranging/ordering	0.59 (0.83)	0.61 (0.83)	0.55 (0.83)	0.693	0.69 (0.92)	0.51 (0.72)	0.218 ^†^
	16. Completeness	0.72 (0.94)	0.76 (0.94)	0.62 (0.96)	0.422	1.06 (1.13)	0.57 (0.82)	0.009 *^,†^
	17. Washing/cleaning	0.30 (0.62)	0.31 (0.64)	0.26 (0.54)	0.652	0.31 (0.54)	0.28 (0.57)	0.758
	18. Checking	0.16 (0.45)	0.18 (0.47)	0.12 (0.40)	0.466	0.23 (0.55)	0.13 (0.41)	0.267 ^†^
	19. Counting	0.09 (0.35)	0.09 (0.34)	0.10 (0.37)	0.925	0.12 (0.43)	0.06 (0.29)	0.370
	20. Hoarding/saving	0.50 (0.85)	0.43 (0.79)	0.69 (0.98)	0.124 ^†^	0.73 (1.10)	0.34 (0.61)	0.021 *^,†^
	21. Repeating	0.45 (0.80)	0.47 (0.83)	0.40 (0.73)	0.638	0.67 (1.00)	0.33 (0.59)	0.026 *^,†^
	24. Sleeping/Bedtime	0.77 (0.99)	0.82 (1.02)	0.62 (0.88)	0.855	1.12 (1.10)	0.60 (0.90)	0.006 *^,†^
	25. Self-care (bathroom and dressing)	0.47 (0.75)	0.45 (0.71)	0.52 (0.86)	0.130	0.58 (0.87)	0.45 (0.70)	0.338
	29. Insists that things remain in the same place(s)	0.61 (0.85)	0.65 (0.88)	0.50 (0.77)	0.325	0.75 (0.97)	0.55 (0.78)	0.223 ^†^
**Ritualistic/Sameness**	26. Travel/transportation	0.46 (0.79)	0.47 (0.81)	0.43 (0.74)	0.754	0.65 (0.97)	0.39 (0.70)	0.064 ^†^
	27. Play/leisure	0.51 (0.83)	0.54 (0.87)	0.43 (0.74	0.444	0.77 (1.00)	0.39 (0.70)	0.018 *^,†^
	30. Objects to visiting new places	0.70 (0.89)	0.76 (0.92)	0.55 (0.80)	0.192	1.06 (1.07)	0.54 (0.74)	0.003 *^,†^
	31. Becomes upset if interrupted in what he/she is doing	0.77 (0.77)	0.77 (0.77)	0.79 (0.78)	0.899	1.12 (0.83)	0.64 (0.71)	0.001 *
	32. Insists on walking in a particular pattern	0.11 (0.37)	0.12 (0.37)	0.10 (0.37)	0.758	0.21 (0.54)	0.06 (0.24)	0.060 ^†^
	33. Insists on sitting in the same place	0.43 (0.68)	0.41 (0.65)	0.48 (0.77)	0.599	0.60 (0.85)	0.37 (0.60)	0.102 ^†^
	34. Dislikes changes in appearance or behavior of the other people around him/her	0.46 (0.74)	0.53 (0.78)	0.29 (0.60)	0.044 *^,†^	0.58 (0.78)	0.45 (0.77)	0.338
	35. Insists on using a particular door	0.17 (0.50)	0.17 (0.52)	0.17 (0.43)	0.960	0.29 (0.64)	0.13 (0.44)	0.129 ^†^
	37. Resists changing activities; difficulty with transitions	1.28 (0.96)	1.41 (0.95)	0.95 (0.91)	0.008 *	1.78 (0.81)	1.05 (0.95)	0.000 *
	38. Insists on the same routine, household, school or work schedule everyday	0.79 (0.93)	0.87 (0.97)	0.57 (0.77)	0.072	1.12 (1.04)	0.62 (0.83)	0.005 *^,†^
	39. Insists that specific things take place at specific times	0.55 (0.83)	0.56 (0.84)	0.50 (0.80)	0.673	0.71 (0.94)	0.46 (0.76)	0.087

*Note.* * = *p* < 0.05, ^†^ = Levene’s Test for Equality of Variances < 0.05 and equal variances not assumed *p*-value reported.

**Table 4:** Repetitive Behavior Scale-Revised (RBS-R) subscale means by overall sample, gender, and ASD diagnostic status according to Bishop et al.’s (2013) 5-factor structure.

RBS-R Subscales by Bishop et al. (2013)	Mean Score Males (SD) (*N* = 112)	Mean Score Females (SD) (*N* = 42)	Mean Differences between Males and Females	Mean Score ASD Diagnosis (SD) (*N* = 52)	Mean Score No ASD Diagnosis (SD) (*N* = 83)	Mean Differences between ASD Diagnosis
**Sensory Motor**	5.37 (4.30)	2. 57 (3.64)	0.000 *^,†^	7.56 (4.82)	3.07 (3.02)	0.000 *^,†^
**Restricted Interests**	2.21 (1.91)	1.57 (1.89)	0.643	3.06 (1.95)	1.47 (1.66)	0.000 *
**Self-injury**	2.77 (3.42)	2.31 (2.71)	0.436	3.67 (3.25)	1.92 (2.44)	0.001 *^,†^
**Compulsive**	4.76 (4.44)	4.38 (5.25)	0.655	6.25 (5.93)	3.81 (3.76)	0.010 *^,†^
**Ritualistic/Sameness**	6.11 (5.17)	4.81 (5.31)	0.170	8.19 (5.86)	4.69 (4.62)	0.000 *

*Note.* * = *p* < 0.05, ^†^ = Levene’s Test for Equality of Variances <0.05 and equal variances not assumed *p*-value reported.

## References

[1] American Psychiatric Association (2013). Diagnostic and Statistical Manual of Mental Disorders.

[2] Oakes A., Thurman A.J., Mcduffie A., Bullard L.M., Hagerman R.J., Abbeduto L. (2016). Characterising repetitive behaviours in young boys with fragile X syndrome. J. Intellect. Disabil. Res..

[3] Wolff J.J., Bodfish J.W., Hazlett H.C., Lightbody A.A., Reiss A.L., Piven J. (2012). Evidence of a distinct behavioral phenotype in young boys with fragile x syndrome and autism. J. Am. Acad. Child. Adolesc. Psychiatry.

[4] Moskowitz L.J., Will E.A., Conner J.B., Black J., Roberts J.E. (2020). Restricted and Repetitive Behaviors in Males and Females with Fragile X Syndrome: Developmental Trajectories in Toddlers Through Young Adults Developmental Trajectories of Restricted and Repetitive Behaviors in Males and Females with Fragile X Syndrome. J. Autism Dev. Disord..

[5] Hagerman R.J., Hagerman P.J. (2002). Fragile X Syndrome: Diagnosis, Treatment, and Research.

[6] Bailey D.B., Raspa M., Olmsted M., Holiday D.B. (2008). Co-occurring conditions associated with FMR1 gene variations: Findings from a national parent survey. Am. J. Med. Genet. Part A.

[7] Cordeiro L., Ballinger E., Hagerman R., Hessl D. (2011). Clinical assessment of DSM-IV anxiety disorders in fragile X syndrome: Prevalence and characterization. J. Neurodev. Disord..

[8] Ciaccio C., Fontana L., Milani D., Tabano S., Miozzo M., Esposito S. (2017). Fragile X syndrome: A review of clinical and molecular diagnoses. Ital. J. Pediatr..

[9] Tassone F., Iong K.P., Tong T.H., Lo J., Gane L.W., Berry-Kravis E., Nguyen D., Mu L.Y., Laffin J., Bailey D.B. (2012). FMR1 CGG allele size and prevalence ascertained through newborn screening in the United States. Genome Med..

[10] Hunter J., Rivero-Arias O., Angelov A., Kim E., Fotheringham I., Leal J. (2014). Epidemiology of fragile X syndrome: A systematic review and meta-analysis. Am. J. Med. Genet. Part A.

[11] Schaefer G.B., Mendelsohn N.J. (2013). Clinical genetics evaluation in identifying the etiology of autism spectrum disorders: 2013 guideline revisions. Genet. Med..

[12] Kaufmann W.E., Kidd S.A., Andrews H.F., Budimirovic D.B., Esler A., Haas-Givler B., Stackhouse T., Riley C., Peacock G., Sherman S.L. (2017). Autism spectrum disorder in fragile X syndrome: Cooccurring conditions and current treatment. Pediatrics.

[13] Klusek J., Martin G.E., Losh M. (2014). Consistency between research and clinical diagnoses of autism among boys and girls with fragile X syndrome. J. Intellect. Disabil. Res..

[14] Talisa V.B., Boyle L., Crafa D., Kaufmann W.E. (2014). Autism and anxiety in males with fragile X syndrome: An exploratory analysis of neurobehavioral profiles from a parent survey. Am. J. Med. Genet. Part A.

[15] Abbeduto L., McDuffie A., Thurman A.J. (2014). The fragile x syndrome-autism comorbidity: What do we really know?. Front. Genet..

[16] Hall S.S., Lightbody A.A., Hirt M., Rezvani A., Reiss A.L. (2010). Autism in fragile X syndrome: A category mistake?. J. Am. Acad. Child. Adolesc. Psychiatry.

[17] Lee M., Martin G.E., Berry-Kravis E., Losh M. (2016). A developmental, longitudinal investigation of autism phenotypic profiles in fragile X syndrome. J. Neurodev. Disord..

[18] Richards C., Jones C., Groves L., Moss J., Oliver C. (2015). Prevalence of autism spectrum disorder phenomenology in genetic disorders: A systematic review and meta-analysis. Lancet Psychiatry.

[19] Evans D.W., Leckman J.F., Carter A., Reznick J.S., Henshaw D., King R.A., Pauls D. (1997). Ritual, Habit, and Perfectionism: The Prevalence and Development of Compulsive-like Behavior in Normal Young Children. Child. Dev..

[20] Thelen E. (1979). Rhythmical stereotypies in normal human infants. Anim. Behav..

[21] DeLoache J.S., Simcock G., Macari S. (2007). Planes, Trains, Automobiles-and Tea Sets: Extremely Intense Interests in Very Young Children. Dev. Psychol..

[22] Harrop C., McConachie H., Emsley R., Leadbitter K., Green J. (2014). Restricted and repetitive behaviors in autism spectrum disorders and typical development: Cross-sectional and longitudinal comparisons. J. Autism Dev. Disord..

[23] Troyb E., Knoch K., Herlihy L., Stevens M.C., Chen C.M., Barton M., Treadwell K., Fein D. (2016). Restricted and Repetitive Behaviors as Predictors of Outcome in Autism Spectrum Disorders. J. Autism Dev. Disord..

[24] Bodfish J.W., Symons F.J., Parker D.E., Lewis M.H. (2000). Varieties of repetitive behavior in autism: Comparisons to mental retardation. J. Autism Dev. Disord..

[25] Martin G.E., Roberts J.E., Helm-Estabrooks N., Sideris J., Vanderbilt J., Moskowitz L. (2012). Perseveration in the connected speech of boys with fragile X syndrome with and without autism spectrum disorder. Am. J. Intellect. Dev. Disabil..

[26] Hall S.S., Lightbody A.A., Reiss A.L. (2008). Compulsive, self-injurious, and autistic behavior in children and adolescents with fragile X syndrome. Am. J. Ment. Retard..

[27] Moss J., Oliver C., Arron K., Burbidge C., Berg K. (2009). The prevalence and phenomenology of repetitive behavior in genetic syndromes. J. Autism Dev. Disord..

[28] Lee A.W., Ventola P., Budimirovic D., Berry-Kravis E., Visootsak J. (2018). Clinical development of targeted fragile X syndrome treatments: An industry perspective. Brain Sci..

[29] McDuffie A., Oakes A., Machalicek W., Ma M., Bullard L., Nelson S., Abbeduto L. (2016). Early language intervention using distance video-teleconferencing: A pilot study of young boys with fragile X syndrome and their mothers. Am. J. Speech Lang. Pathol..

[30] Crawford H., Moss J., Stinton C., Singla G., Oliver C. (2018). Overactivity, impulsivity and repetitive behaviour in males with fragile X syndrome: Contrasting developmental trajectories in those with and without elevated autism symptoms. J. Intellect. Disabil. Res..

[31] McDuffie A., Abbeduto L., Lewis P., Kover S., Kim J.S., Weber A., Brown W.T. (2010). Autism spectrum disorder in children and adolescents with fragile X syndrome: Within-syndrome differences and age-related changes. Am. J. Intellect. Dev. Disabil..

[32] Sherman S.L., Kidd S.A., Riley C., Berry-Kravis E., Andrews H.F., Miller R.M., Lincoln S., Swanson M., Kaufmann W.E., Brown W.T. (2017). Forward: A registry and longitudinal clinical database to study fragile X syndrome. Pediatrics.

[33] Roid G.H. (2003). Stanford-Binet Intelligence Scales.

[34] Sansone S.M., Schneider A., Bickel E., Berry-Kravis E., Prescott C., Hessl D. (2014). Improving IQ measurement in intellectual disabilities using true deviation from population norms. J. Neurodev. Disord..

[35] Aman M.G., Singh N.N., Stewart A.W., Field C.J. (1985). The aberrant behavior checklist: A behavior rating scale for the assessment of treatment effects. Am. J. Ment. Defic..

[36] Rutter M., Bailey A., Lord C. (2003). Social Communication Questionnaire.

[37] Constantino J., Gruber C. (2012). Social Responsiveness Scale.

[38] Sansone S.M., Widaman K.F., Hall S.S., Reiss A.L., Lightbody A., Kaufmann W.E., Berry-Kravis E., Lachiewicz A., Brown E.C., Hessl D. (2012). Psychometric study of the aberrant behavior checklist in fragile X syndrome and implications for targeted treatment. J. Autism Dev. Disord..

[39] Scahill L., Aman M.G., Lecavalier L., Halladay A.K., Bishop S.L., Bodfish J.W., Grondhuis S., Jones N., Horrigan J.P., Cook E.H. (2015). Measuring repetitive behaviors as a treatment endpoint in youth with autism spectrum disorder. Autism.

[40] Mirenda P., Smith I.M., Vaillancourt T., Georgiades S., Duku E., Szatmari P., Bryson S., Fombonne E., Roberts W., Volden J. (2010). Validating the repetitive behavior scale-revised in young children with autism spectrum disorder. J. Autism Dev. Disord..

[41] Lam K.S.L., Aman M.G. (2007). The repetitive behavior scale-revised: Independent validation in individuals with autism spectrum disorders. J. Autism Dev. Disord..

[42] Bishop S.L., Hus V., Duncan A., Huerta M., Gotham K., Pickles A., Kreiger A., Buja A., Lund S., Lord C. (2013). Subcategories of restricted and repetitive behaviors in children with autism spectrum disorders. J. Autism Dev. Disord..

[43] Hooker J.L., Dow D., Morgan L., Schatschneider C., Wetherby A.M. (2019). Psychometric analysis of the repetitive behavior scale-revised using confirmatory factor analysis in children with autism. Autism Res..

[44] Berry-Kravis E., Hessl D., Abbeduto L., Reiss A.L., Beckel-Mitchener A., Urv T.K., Measures O., Groups W., Aman M., Clapp K. (2013). Outcome Measures for Clinical Trials in Fragile X Syndrome. J. Dev. Behav. Pediatr..

[45] Yatawara C.J., Einfeld S.L., Hickie I.B., Davenport T.A., Guastella A.J. (2016). The effect of oxytocin nasal spray on social interaction deficits observed in young children with autism: A randomized clinical crossover trial. Mol. Psychiatry.

[46] Bernaerts S., Boets B., Bosmans G., Steyaert J., Alaerts K. (2020). Behavioral effects of multiple-dose oxytocin treatment in autism: A randomized, placebo-controlled trial with long-term follow-up. Mol. Autism.

[47] Hardan A.Y., Fung L.K., Libove R.A., Obukhanych T.V., Nair S., Herzenberg L.A., Frazier T.W., Tirouvanziam R. (2012). A randomized controlled pilot trial of oral N-acetylcysteine in children with autism. Biol. Psychiatry.

